# Sonographic Wrist Measurements and Detection of Anatomical Features in Carpal Tunnel Syndrome

**DOI:** 10.1155/2014/657906

**Published:** 2014-02-04

**Authors:** Esther Vögelin, Thomas Mészàros, Franziska Schöni, Mihai A. Constantinescu

**Affiliations:** ^1^Department of Plastic and Hand Surgery, University Hospital, Inselspital, University of Bern, Freiburgstraße, 3010 Bern, Switzerland; ^2^Etude Suisse de Cohorte VIH, CHUV, Bipôle 2, Route de la Corniche 10, 1010 Lausanne, Switzerland

## Abstract

*Introduction*. This study compares anatomical findings at wrist level in patients with known carpal tunnel syndrome (CTS) and controls by ultrasonography (US). *Material and Methods*. Wrist-US investigations of 28 consecutive patients with 38 diagnosed, idiopathic CTS were compared to 49 healthy volunteers without history of CTS. Internal wrists dimensions, the presence of flexor muscle bellies in the carpal tunnel, and cross-sectional area of the median nerve were analyzed. The findings were correlated to gender, age, and BMI. *Results*. US demonstrated a square internal carpal tunnel configuration in CTS patients compared to controls (*P* < 0.001). Patients with CTS showed a trend towards the presence of flexor muscles bellies in the carpal tunnel (odds ratio 1.77, 95% CI 0.337–8.33). CTS was present in women with higher BMI (*P* = 0.015). *Conclusion*. US allowed detection of specific anatomical features at wrist level in CTS patients. This observation may enable—following confirmation in larger prospective studies—risk evaluation for CTS development.

## 1. Introduction 

Carpal tunnel syndrome (CTS) reflects the most common form of peripheral nerve entrapment [1]. Diagnosis of CTS is based on clinical symptoms and usually confirmed by nerve conduction studies. In recent years, the value of sonography (US) has been evaluated for the assessment of both median nerve and carpal tunnel pathology [2–4]. Sonographic measurement of the median nerve cross-sectional area (CSA) is both sensitive (89%) and specific (83%) for the diagnosis of CTS [5]. Recently, the sensitivity and specitivity could be improved by introduction of the median nerve CSA ratio [6]. The wrist-to-forearm ratio of the median nerve CSA was introduced to exclude variations in different populations and measurement techniques allowing therefore patients to serve as their own control [6, 7].

In addition to isolated observations of the median nerve, US allows noninvasive dynamic observations of other anatomical structures passing the carpal tunnel. In a cadaveric study, 46% of the investigated female bodies were reported to exhibit flexor muscles in the proximal carpal tunnel during extension of the fingers compared to only 7.8% of men [8]. The presence of muscle bellies entering the carpal tunnel may increase the pressure during wrist and finger extension [9]. Furthermore, personal factors such as age, body mass index (BMI), and hand anthropometric measurements have been reported to be risk factors for CTS development [10, 11]. Higher wrist and hand shape indices seem to be independent risk factors in females but not in males [10]. Finally, Kamolz et al. [12] demonstrated that patients with CTS have a more quadratic wrist and carpal tunnel shape as well as a shorter hand configuration compared to controls. However, they did not correlate their findings to gender [12]. Recently, ultrasound findings in CTS have been extensively studied, showing difference in median nerve characteristics (mainly the cross-sectional area), but also in anatomy of the carpal tunnel especially demonstrating the movements of structures within the carpal tunnel. However, the correlation of anatomical wrist configuration in combination with the median nerve surrounding muscles has not yet been addressed.

This prospective clinical study investigates in vivo anatomical wrist and carpal tunnel configurations by ultrasonography (US) and evaluates statistically the differences between diagnosed CTS patients and healthy controls.

We hypothesize the presence of particular anatomical wrist configurations (internal wrist ratio, prevalence of muscle bellies, etc.) to be detectable by US in CTS patients and to be statistically different from healthy controls.

## 2. Material and Methods

Twenty-eight consecutive patients undergoing treatment for diagnosed idiopathic CTS and 49 healthy volunteers without signs of CTS were included in this sonographic quality control study. This investigation was approved by the Institutional Review Board of University of Bern in accordance with the rules and regulations for quality control studies. Informed consent was obtained for the sonographic data analysis of all patients. The electrophysiological findings were considered pathological compared to the reference standard of the following age dependent norm values for sensory conduction velocity (<41–53 m/second), distal motor latency (>3.9–4.1 msec), and muscle action potential (<5 mV). Exclusion criteria for CTS patients were sustained trauma to the upper extremity, rheumatoid arthritis, hypothyroidism, or other conditions known to induce CTS. All volunteers included in the control group of the study displayed no clinical signs, symptoms, or history of CTS. Electrodiagnostic tests were not performed in the healthy volunteers.

Age and gender were recorded and each subject's body mass index (BMI) was calculated.

Both controls and patients underwent an ultrasound examination of the carpal tunnel using a 5–12 MHz linear array transducer (Philips En Visor Medical System, Bothell, WA). Subjects were seated in a chair with their arms extended and hands resting in a horizontal supine position on an examination couch, with fingers semiextended [13]. Sonographic evaluation was performed by one ultrasound trained investigator (TM).

### 2.1. Sonographic Measurements

**Figure 1:**
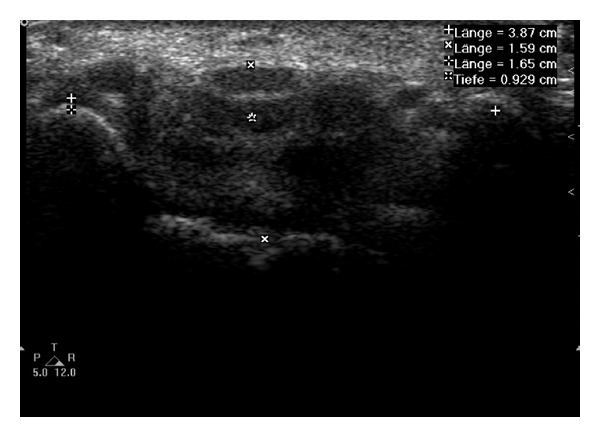
Internal wrist measurement: the radio-ulnar (width) distance between the pisiform (+) and scaphoid tubercle (+) and the dorsopalmar (depth) distance between the flexor retinaculum above the median nerve (*x*) and the lunate bone (*x*) were measured sonographically in the carpal tunnel.

To measure the *internal carpal tunnel dimensions* (Figure 1), the radio-ulnar diameter and the dorsopalmar diameter of the carpal tunnel were measured at defined levels between the scaphoid and the pisiform bone, the flexor retinaculum above the median nerve, and the lunate bone, respectively. The internal carpal tunnel ratio was calculated dividing width by depth.

**Figure 2:**
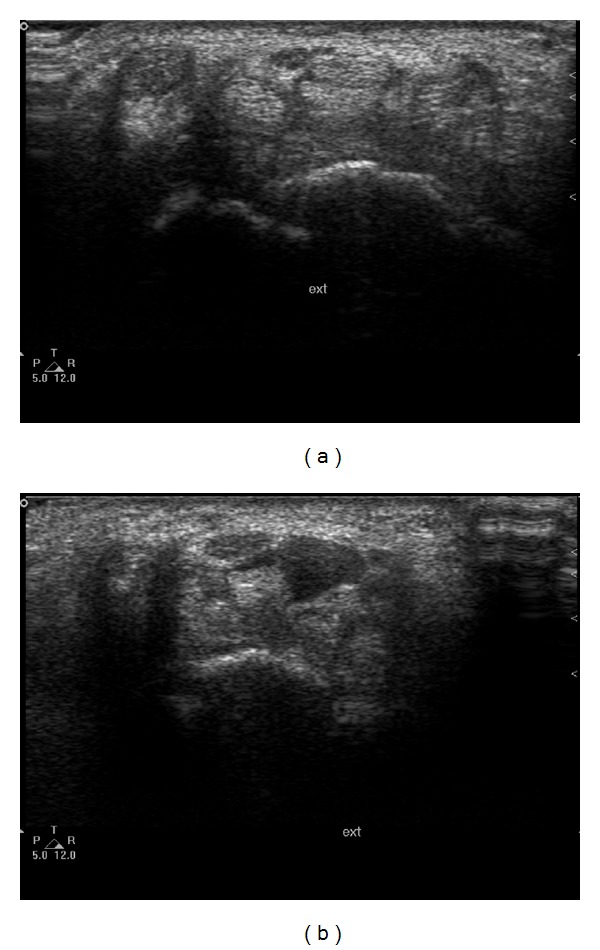
Presence of flexor muscles at the entrance of the carpal tunnel. Median nerve and flexor tendons without muscles (a), Median nerve and flexor tendons with muscles (b).

The *prevalence of FDS and FDP muscle bellies* during the full range of motion of the fingers was determined at the entrance of the carpal tunnel (line between the scaphoid tubercle and pisiform bone) (Figures 2(a) and 2(b)). With the fingers in extended position, the presence of muscle bellies was confirmed by demonstrating the typical hypoechoic muscle pattern between the tendons and the median nerve at the entrance of the carpal tunnel.

The wrist-forearm ratio was calculated by dividing the distal cross-sectional area (CSA) of median nerve by the proximal CSA of median nerve [14]. To this end, the distal CSA was determined as the largest CSA of the median nerve (cm^2^) within the carpal tunnel using manual tracing in serial transversal scans [13]. The proximal CSA of the median nerve was obtained by direct tracing method 2 cm proximal of the main wrist crease fold where the nerve is located superficially beyond the forearm fascia.

All sonographic wrist measurements were performed by the same investigator (TM). The wrist-forearm ratio including the proximal and distal CSA of the median nerve was calculated perioperatively within 6 weeks of carpal tunnel release. Delayed postoperative measurements were not performed since it is known that the cross-sectional area of the median nerve changes significantly after 3 months following carpal tunnel release [13]. In bilaterally treated patients, a scar was visible following previous surgery, wherefore the examiner could not be blinded to the group. For the statistical analysis models, the measurements of the dominant hand were used in patients who presented bilateral CTS.

### 2.2. Statistical Methods

The investigation included descriptive statistics of means or medians, depending on the distribution of the data and standard deviations or range, respectively. Nonparametric tests were used to compare means. Logistic regression models were used for the multivariate analysis including age, gender, BMI, and all sonographic wrist measurements. SAS 9.2 (SAS Institute Inc., Cary, NC, USA) was used for all analyses and the level of significance was set at 0.05 throughout the study.

## 3. Results

The 28 patients had a mean age of 56 years (16 men: 56 ± 16.4 years, 12 women: 56.2 ± 13.5 years). The female patients presented with 4 bilateral and 8 unilateral CTS and the male patients presented with 6 bilateral and 10 unilateral CTS resulting in a total of 38 operated CTS. The healthy control population had a mean age of 53 years (24 women: 50.5 ± 14.5 years and 25 men: 52.5 ± 9.9 years) (Table 1).

There was no significant difference in age, distribution of right and left wrists, or dominance between the patients and the healthy volunteers. A statistically significantly higher BMI was found in female but not in male CTS patients compared to the control group (female patients: 28.6 ± 4.2; female control: 25.1 ± 5.8, *P* = 0.015; male patients: 26.8 ± 4.6; male control: 28.2 ± 6.5, *P* = 0.64).

### 3.1. Sonographic Measurements

#### 3.1.1. Internal Carpal Tunnel Ratio of the Width/Depth

The internal dimensions of the carpal tunnel with the width/depth ratio indicate the shape of the carpal tunnel (square versus rectangular) and showed a more square shaped configuration in the CTS group compared to the control group. There was a highly significant difference of the mean internal carpal tunnel ratio between male patients and controls (male patients: 0.42 ± 0.05; male controls: 0.34 ± 0.04, *P* = 0.0002) as well as between female patients and controls (female patients: 0.39 ± 0.06; female controls: 0.35 ± 0.04, *P* = 0.034).

#### 3.1.2. The Prevalence of Flexor Muscle Bellies

Patients with CTS showed a slightly higher presence of flexor muscles bellies in the carpal tunnel (odds ratio 1.77) (95% CI 0.337–8.33, *P* value 0.52), which however did not reach statistical significance in this sample. Muscle bellies were present at the carpal tunnel entrance between the pisiform and scaphoid tubercle in 29 (14 males, 15 females) of 38 treated CTS. In contrast, 34 (14 males, 20 females) of 49 controls demonstrated muscle bellies at the carpal tunnel inlet during extension. (Table 2) The presence of muscle bellies was independent of internal width/depth ratio or BMI when corrected for age and sex in both groups.

#### 3.1.3. The Cross-Sectional Area (CSA) of Median Nerve (cm^2^)

The mean distal CSA of the median nerve at the carpal entrance between scaphoid and pisiform bone in men (male patients: 0.13 cm^2^ ± 0.04; male controls: 0.11 cm^2^ ± 0.04, *P* = 0.0019) and women (female patients: 0.12 cm^2^ ± 0.03; female controls: 0.10 cm^2^ ± 0.03, *P* = 0.0424) with CTS was significantly larger compared to the control group. The mean proximal CSA of the median nerve was 0.099 cm^2^ ± 0.017 in the male patients with CTS and 0.086 cm^2^ ± 0.016 in the healthy volunteers demonstrating a slightly larger median nerve CSA in patients than controls (*P* = 0.0348). The same trend could be seen in the mean proximal CSA of female patients (0.10 cm^2^ ± 0.029) and controls (0.096 cm^2^ ± 0.066) but did not achieve significant differences in this comparison (*P* = 0.17).

The ratio of the CSA of the median nerve (distal/proximal CSA in cm^2^) between male patients and controls was statistically significant (*P* = 0.036) showing a higher ratio in CTS patients caused by a larger distal CSA of the median nerve. However, this ratio did not reach statistical significance in female patients compared to controls (*P* = 0.2) (Table 3).

**Figure 3:**
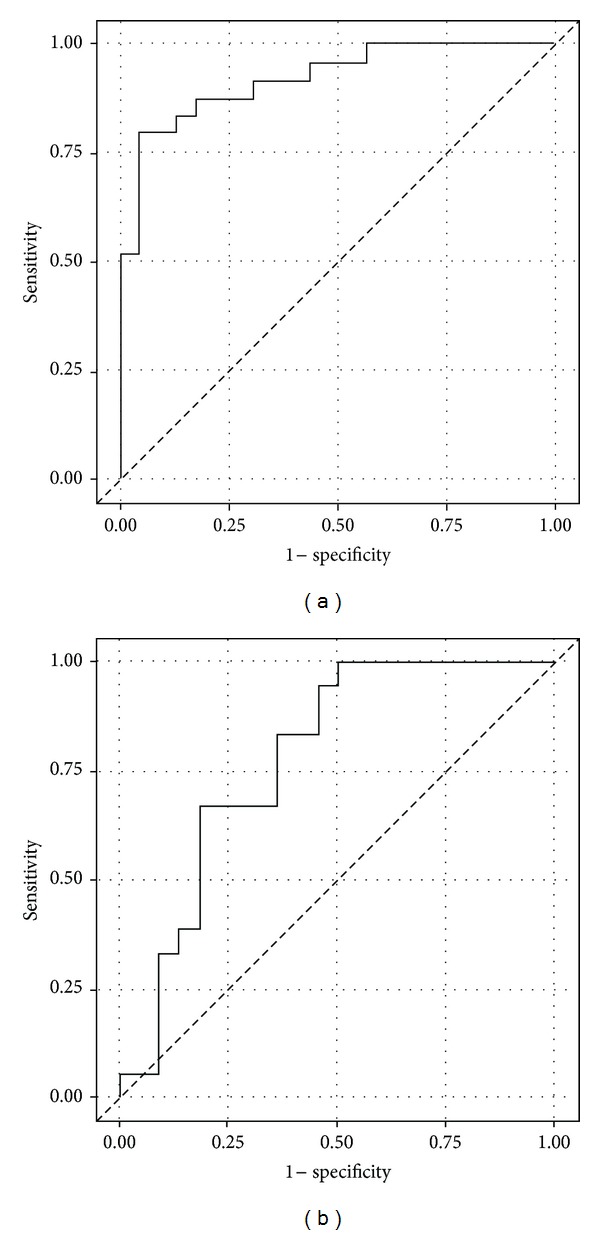
The internal carpal tunnel ratio (inner ratio) is plotted against the distal CSA. The squarer the carpal tunnel shape (numbers towards values of 1) and the higher the CSA value are, the more likely to have or to develop CTS. In male patients, the cutoff between patients and controls follows a distinct descending line (ROC area under the curve = 0.9235; CI 0.85 to 0.997, *P* < 0.0001) (a). In females the cutoff line is less distinct (ROC area under the curve = 0.7778; CI 0.63 to 0.93 *P* = 0.0042) (b). *R*-squared values are in male patients: 0.039 and in controls: 0.004, in female patients: 0.024 and in female controls: 0.060.

The plot of inner ratio to the distal CSA of the median nerve (Figure 3) shows that those subjects with more square shaped carpal tunnels are more likely to develop CTS. Additionally, the distal CSA seems to be bigger in the diseased subjects. However, one cannot distinguish if the higher distal CSA is a consequence of more square shaped carpal tunnel. In male patients the cutoff between patients and controls follows a distinct descending line (ROC area under the curve = 0.9235; CI 0.85 to 0.997, *P* < 0.0001) (Figure 3(a)). In females the cut-off line is less distinct (ROC area under the curve = 0.7778; CI 0.63 to 0.93 *P* = 0.0042) (Figure 3(b)).

## 4. Discussion 

Do anatomical factors in the carpal tunnel predispose CTS development?

This question has been frequently raised but never entirely answered. The current study focused on local anatomical features at wrist level using noninvasive ultrasound imaging. By means of this technique, statistically significant differences in anatomical wrist configurations were observed (internal wrist ratio, shape of the carpal tunnel) between CTS patients and healthy controls. Based on the results of this study, noninvasive sonographic detection of statistically significant local anatomical factors at wrist level may allow further detection and validation of CTS-pertinent anatomy.

Carpal tunnel syndrome has been associated with several personal risk factors such as age, sex, BMI, and external wrist dimensions [10, 11]. Over the past two decades, sonography has been used as an additional diagnostic tool for CTS detection [3, 15]. The results of the current study including *internal wrist measurements* demonstrated both men and women suffering from CTS to have a significantly squarer shape of the carpal tunnel (width/depth ratio) and a larger CSA of the median nerve at the carpal tunnel entrance (wrist/forearm ratio) when compared with healthy controls who display a more rectangular carpal tunnel shape. Especially, in patients with negative electrodiagnostic findings such combinations of sonographic anatomical findings may be helpful in diagnosing CTS [16]. In contrast to other reports, the BMI in CTS patients versus controls showed only statistically significant differences in women and not in men [10, 11].

*The CSA of the median nerve* has proven to be valid in diagnosing CTS. Prior studies have proposed a range of distal median nerve CSA cutoff values [3, 4, 15, 17]. In order to achieve diagnostic independence of interindividual variability of the CSA of the median nerve at wrist level alone, investigators calculated the CSA ratio of the median nerve at wrist and forearm level [6, 7, 14].

Our findings parallel the reported statistically significant differences of larger distal CSA compared to proximal CSA measured at different levels in the forearm in men and showed the same trend in women however without reaching statistical significance. Explanations could be the small sample size of female CTS patients, the lack of correlation of the severity of CTS between electrodiagnostic studies and sonographic measurements, or the level for the proximal measured CSA which may have been chosen too distal thus being still in the area of the median nerve swelling.

Some studies have shown that the sensitivity of the wrist-forearm median nerve ratio for CTS is higher than median nerve CSA at the wrist alone while other studies contradict these observations [18]. The use of ratios is a helpful element in eliminating issues of variability between populations, as the patients become their own individual control. In this study, *the internal carpal tunnel ratio* discriminated well between CTS patients and controls (cutoff at 0.385). Additional discrimination can be achieved by the use of two variables instead of one. Here logistic regression of the sonographic measurements at wrist level revealed a significant risk of CTS when relying on both variables internal carpal tunnel ratio (width/depth) and distal CSA of the median nerve, after controlling for age and gender. Nevertheless, one should consider the possibility of the observed increased CSA to be a reaction rather than the cause of the constricted space in a square shaped carpal tunnel.

The analysis of *prevalence of flexor muscles* in the carpal tunnel between CTS patients and healthy controls did not reveal any different distribution for both genders. Although flexor muscles bellies were more frequently present in women (patients and controls) than in men at carpal tunnel level, this finding did not reach statistical significance. In the current literature, only one anatomical cadaver study demonstrated a higher prevalence of flexor muscles in women than, men [8]. This interesting trend may prove in future larger studies by means of noninvasive US investigation that the presence of flexor muscle bellies within the carpal tunnel does increase the statistical risk of CTS development.

In recent studies, median nerve motion and displacement between tendons have been analysed during independent long-finger flexion and fist motion in healthy volunteers [19]. The reduced space in squarer wrist shapes might cause changing of the connective gliding tissue to fibrosis and therefore the presence of muscles could be anticipated as a promoter of CTS. Furthermore, with US a reduced longitudinal gliding of the median nerve at the wrist in CTS patients has been demonstrated [20, 21] indicating the fibrotic changes of the gliding tissue in the too tight canal.

In summary, muscle prevalence and muscle movement in the carpal canal in combination with a square carpal tunnel form and an increased BMI in women appear to favour the development of CTS.

The fact that electrodiagnostic tests were not performed in the healthy volunteers of this study must be considered to be a limitation. In order to account for this limitation each volunteer was questioned specifically about any signs of CTS in his past history and underwent a clinical examination prior to the ultrasound measurements. The limited number of patients available for this prospective study may have interfered with the achievement of statistical significance of certain observed trends in the sonographic measurements. Furthermore, the sonographer could not be blinded since some patients presented scars from previous contralateral CTS-releases. Finally, this study has a case-control design similar to most data in the literature. This renders it to some extent difficult to determine if differences in patients predisposed them to CTS or whether the differences are the results of the CTS. On the other hand, some of the presented findings like wrist configuration and shape (internal ratio) and the presence or absence of flexor muscles during flexion and extension of the wrist are unlikely to be caused by the CTS and must therefore be accepted as potentially predisposing anatomical factors.

It is the use of noninvasive ultrasound which enabled both the static and dynamic imaging and allowed the determination and description of particular, so far undetected anatomical configurations of the wrist and median nerve in patients suffering from CTS. To establish a predictive use from the observations made in this study, a larger scale prospective, long-term analysis of healthy individuals will be necessary.

## 5. Conclusion

Ultrasound allowed the detection of statistically significant anatomical wrist features in CTS patients when compared to healthy controls. The observed square internal wrist configuration at carpal tunnel level allows together with the median nerve CSA a statistical correlation with CTS occurrence. The presence of flexor muscle bellies within the carpal tunnel may additionally influence CTS development. Based on the anatomical observation made possible by non-invasive ultrasound imaging in this study, further larger scale prospective evaluations are warranted for life-time risk evaluation for CTS development using this method.

## Figures and Tables

**Table 1:** Number of patients and operations with CTS.

Cases	Site of operation			Total
	Right	Left	Bilateral	
Women	6	2	4	12
Men	8	2	6	16

Total patients	14	4	10	**28**
Total operations	14	4	20	**38**

Bold numbers: number of total operations (more than patients) and number of total patients.

**Table 2:** Prevalence and gender distribution of muscles in patients and controls.

	Prevalence of muscle bellies in carpal tunnel				Total	
	Men		Women			
	Present	Not present	Present	Not present	Present	Not present
Patients	14	8	15	1	29	9
Controls	14	11	20	4	34	15

Total	28	19	35	5		

**Table 3:** Distal and proximal cross-sectional area (CSA) of median nerve including ratio (distal/proximal CSA).

	Male			Female		
	Patients	Controls	*P* value	Patients	Controls	*P* value
Distal CSA (cm^2^)	0.14 ± 0.04	0.10 ± 0.04	**0.0019**	0.12 ± 0.03	0.10 ± 0.03	**0.04**
Proximal CSA (cm^2^)	0.099 ± 0.02	0.087 ± 0.02	**0.02**	0.096 ± 0.02	0.084 ± 0.02	**0.08**
Ratio (distal/proximal CSA)	1.414	1.149	**0.036**	1.25	1.19	**0.2**

*P* values stand for statistical significance looking at CSA distal, proximal, and the ratio between patients and control in female and male.
